# Imaging nucleic acids in biofilms

**DOI:** 10.1042/EBC20250031

**Published:** 2026-09-02

**Authors:** Gabriel Antonio S. Minero, Freja Winther Sillesen, Rikke Louise Meyer

**Affiliations:** 1 Interdisciplinary Nanoscience Center, Gustav Wieds Vej 14, 8000 Aarhus C, Denmark; 2 Department of Biology, Ny Munkegade 114-116, 8000 Aarhus C, Denmark

**Keywords:** biofilm, eDNA, eRNA, Extracellular matrix, fluorescence, imaging

## Abstract

Extracellular nucleic acids (eNA) are ubiquitous components of bacterial biofilms, contributing to structural integrity, antimicrobial resistance, and immune evasion. While traditionally viewed as extracellular DNA in the canonical B-form, recent discoveries reveal remarkable structural diversity within biofilm matrices, including left-handed Z-DNA, G-quadruplexes, i-motifs, triplexes, and extracellular RNA. These non-canonical structures possess emergent properties distinct from B-DNA: they resist degradation by mammalian DNase I, they form catalytically active DNAzymes with peroxidase activity, and they serve as conduits for extracellular electron transfer. Despite their biological significance, these structures have been largely overlooked due to limitations in visualisation methods.

The present review critically evaluates current tools for imaging eNA in biofilms, with emphasis on fluorescent DNA-binding dyes and structure-specific immunolabelling approaches. The widely used DNA-binding dyes, such as propidium iodide and TOTO™-1, exhibit strong structural biases and render non-canonical structures invisible. This has important implications for interpreting nuclease treatment outcomes and biofilm composition. We provide practical guidance for comprehensive eNA visualisation, recommending integration of multiple detection methods: combining far-red dyes with TOTO™-1 for broad structural coverage and validating findings with commercial monoclonal antibodies (Z22, BG4, 1H6, iMab, Jel466, and J2) alongside functional assays. Understanding eNA structural diversity and developing appropriate detection tools are essential for rational design of structure-specific nuclease therapeutics targeting biofilm infections.

## Introduction

Biofilms are highly resilient communities of microorganisms embedded in a self-produced polymeric matrix of polysaccharides, proteins, lipids, and extracellular nucleic acids. This matrix is key to biofilms' resilience, as it protects bacteria against predators and environmental stressors [1]. While the matrix composition is highly variable among different microorganisms, there is one matrix component that seems to be ubiquitous: DNA [2]. Bacteria cannot initiate biofilm formation without extracellular DNA (eDNA) [3], and eDNA impacts the biofilm's mechanical properties [4], resistance to invasion by, e.g., immune cells [5], and tolerance to antibiotics [6]. DNA accumulates in the biofilm matrix as a result of, e.g., explosive cell lysis [7], autolysis [8], prophage activity [9], active secretion through type IV secretion systems [10], and extracellular vesicle formation [11,12]. DNA synthesis can even continue in the extracellular space after bacterial lysis [13]. Moreover, recent research has revealed that biofilms not only contain DNA but also RNA [14], and we should therefore think of extracellular nucleic acids (eNA) rather than eDNA.

**Figure 1:**
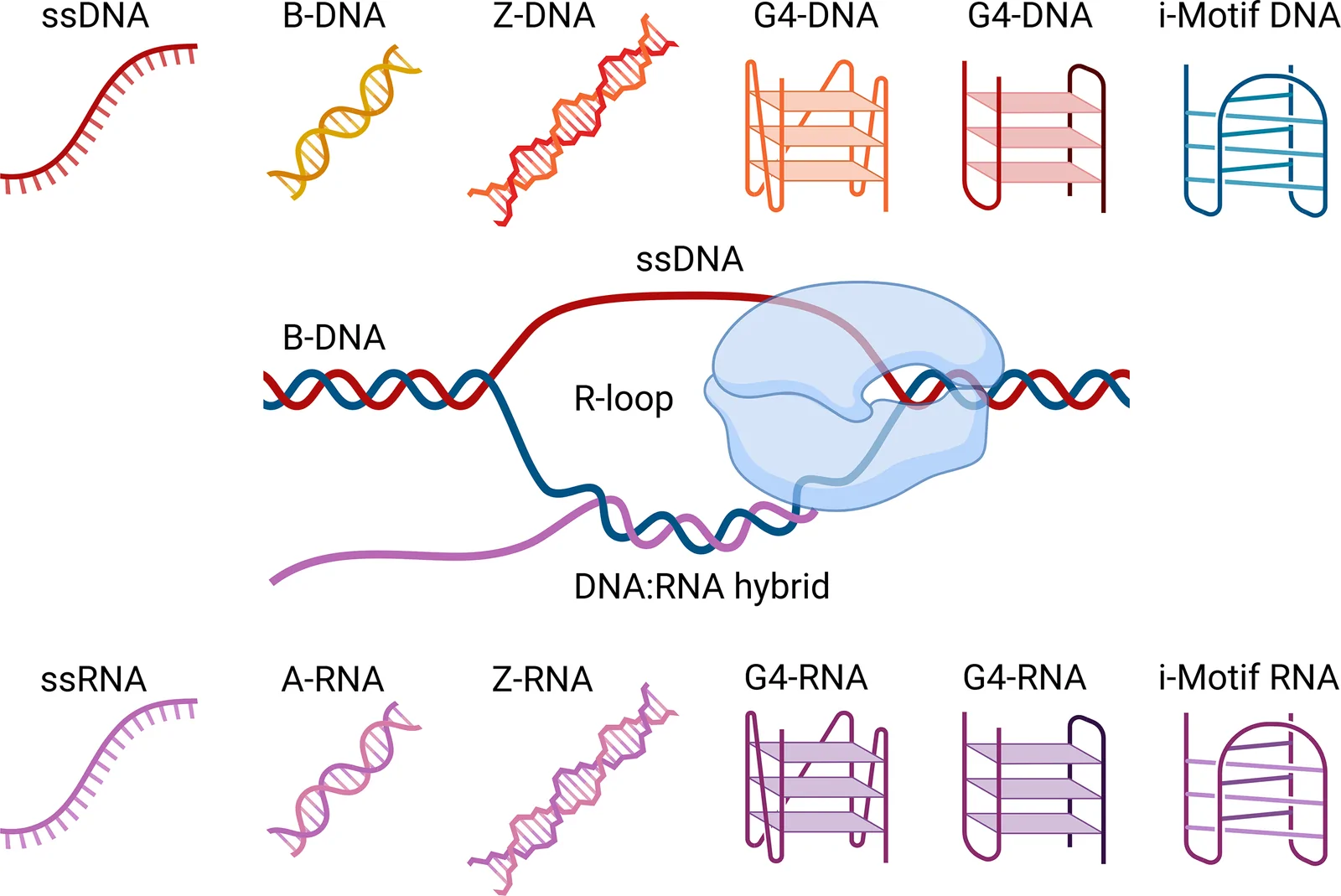
Structures of DNA and RNA detected in biofilms Structures of DNA and RNA detected in biofilms (created in BioRender. Minero, G. A. (2026) https://BioRender.com/a45q416). DNA exists stably in its canonical B-form, consisting of sense (blue) and antisense (red) strands. The dsDNA can fold into a non-canonical left-handed Z-DNA helix. During transcription, ssDNA and a DNA:RNA hybrid form in the transcription loop, and ssDNA can form intra- and intermolecular G4-DNA (red) and i-Motif DNA (blue). Similarly, RNA (magenta) can exist as ssRNA or double-stranded A-RNA, Z-RNA, i-Motif, or intra- and intermolecular G4-RNA (all shown in magenta).

The structure of eNA is also rather diverse (Figure 1). In addition to the canonical single- and double-stranded DNA, biofilms contain the left-handed Z-DNA helix, i-motifs, and G-quadruplexes (Figure 1). These non-canonical nucleic acid structures can form from DNA, RNA, or DNA/RNA hybrids, and they provide emergent properties to biofilms. Firstly, they are resistant to degradation by mammalian DNase I, the enzyme tasked with removing eDNA in the human body [15]. Moreover, G4 and Z-DNA are pro-inflammatory and a potential cause of autoimmunity [16].

Recent studies have identified non-canonical eNA as a therapeutic target for biofilm infections. For example, converting Z-DNA to B-DNA by removing Z-DNA-stabilising bacterial proteins leads to a better therapeutic outcome for biofilm infections [17]. There is also scope for degrading Z-DNA directly, using bacterial and fungal nucleases [18], and the prospect of more effective eNA removal in biofilms opens up new possibilities to develop effective enzyme-based biofilm therapeutics.

G-quadruplexes are particularly interesting due to their impact on biofilm elasticity [19] and their capability of binding hemin to form a peroxidase-like DNAzyme [20]. G4 can act as a conduit for extracellular electron transfer, which facilitates bacterial respiration by transporting electrons to electron acceptors some distance away from the cell [21]. Surface-associated G4 was also shown to be responsible for extracellular electron transfer in methanogenic archaea, enabling electro-methanogenesis where the microorganisms reduce CO_2_ to CH_4_ with electrons from an electrode [22]. The role of eNA in biofilms is thus much more diverse than initially assumed. It is therefore timely to reassess the tools available to visualise extracellular nucleic acids in biofilms and how their sensitivity and specificity enable investigation of eNA beyond the canonical B-DNA helix.

## Visualising eNA with DNA-binding dyes

Visualisation of extracellular nucleic acids in biofilms by fluorescence microscopy started with the application of membrane-impermeable DNA-binding dyes, such as propidium iodide (PI), cyanine monomers (e.g., SYTOX™), or cyanine dimers (e.g., TOTO™). These dyes come in many colours, making them a convenient and fast tool for eDNA imaging. The dyes are positively charged to facilitate interaction with DNA, typically by intercalation, increasing in fluorescence as they bind. The ‘turn on’ properties of the dyes allow sensitive detection and high signal/noise ratio without having to wash the sample. Table 1 summarises the properties of membrane-impermeable dyes that have been used for live/dead staining or eDNA staining in microscopy or flow cytometry. The different classes of dyes vary in quantum yield, photostability, binding affinity, base specificity, and selectivity to DNA or RNA. The selection of dyes therefore balances the need for brightness, stability, specificity, and excitation/emission spectrum.

**Table 1:** Overview of DNA-binding dyes summarising information from the manufacturers and from research into their specificity for different nucleic acid structures and their applicability in biofilms. The structures are redrawn from [32] using Chemdraw. The dyes are sold as light up probes for B-DNA, and we indicate here the fluorescence intensity relative to B-DNA (✓ = similar to B-DNA, ✓✓ = brighter than B-DNA (>300%), ✘ = less than B-DNA (<30 %) when bound to RNA or non-canonical NA: G4-NA (5’-GA GGG T GGG TA GGG T GGG) or Z-NA (5’-GC^Br^GC^Br^GC/3’-C^Br^GC^Br^GCG) according to [30]

Dye	Structure	Quantum yield	Ex/Em, nm	DNA			RNA			Notes
				B	Z	G4	A	Z	G4	
Cyanine dimers										
TOTO, POPO, BOBO, and YOYO series: Pro: High affinity, high fluorescence. Medium photostability. Con: High cost. Strong background from dead cells.										
TOTO™-1	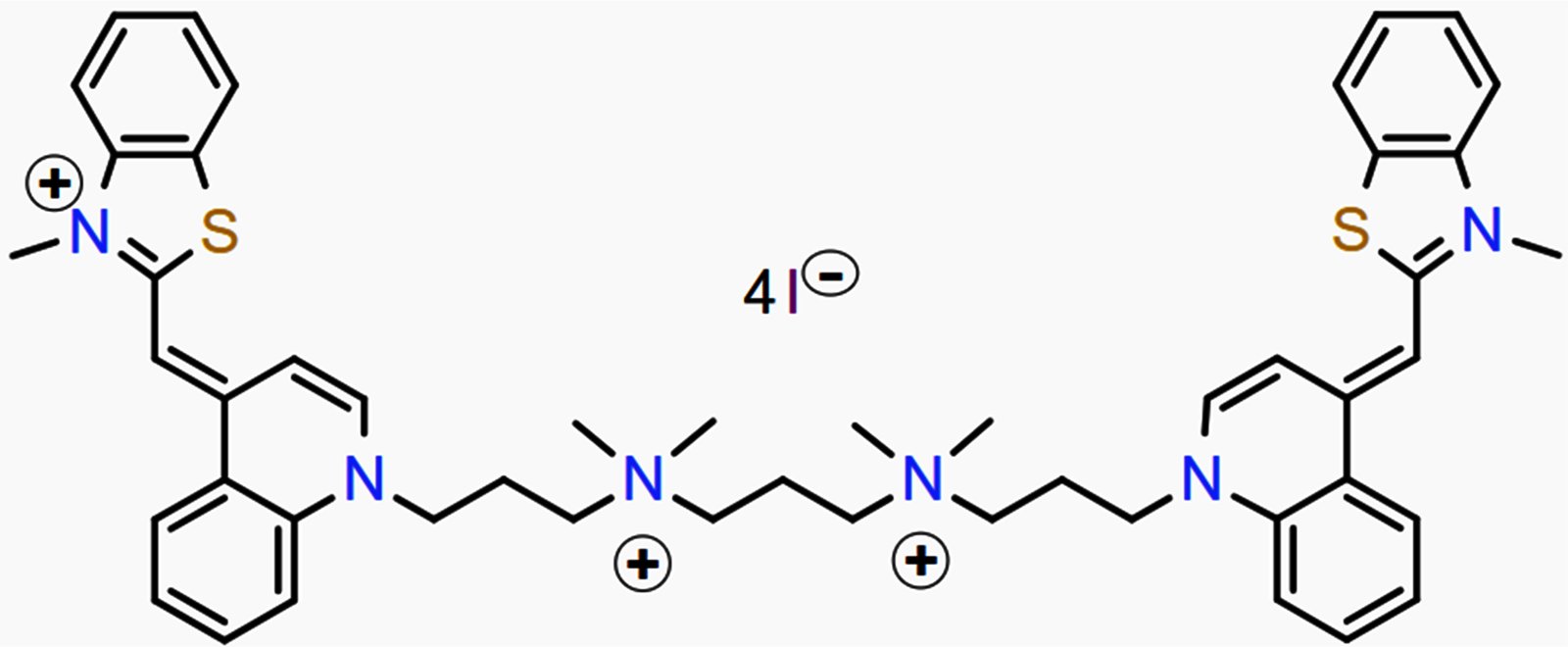	0.34	514/532	√	✘	✘	√	✘	✘	Widely used in biofilm research [14,33,34]. Highly specific for B-DNA and has some preference for CTAG regions [35]. Has also been used in combination with SYTO60 to form a FRET pair to detect G4 [36].
TOTO™-3	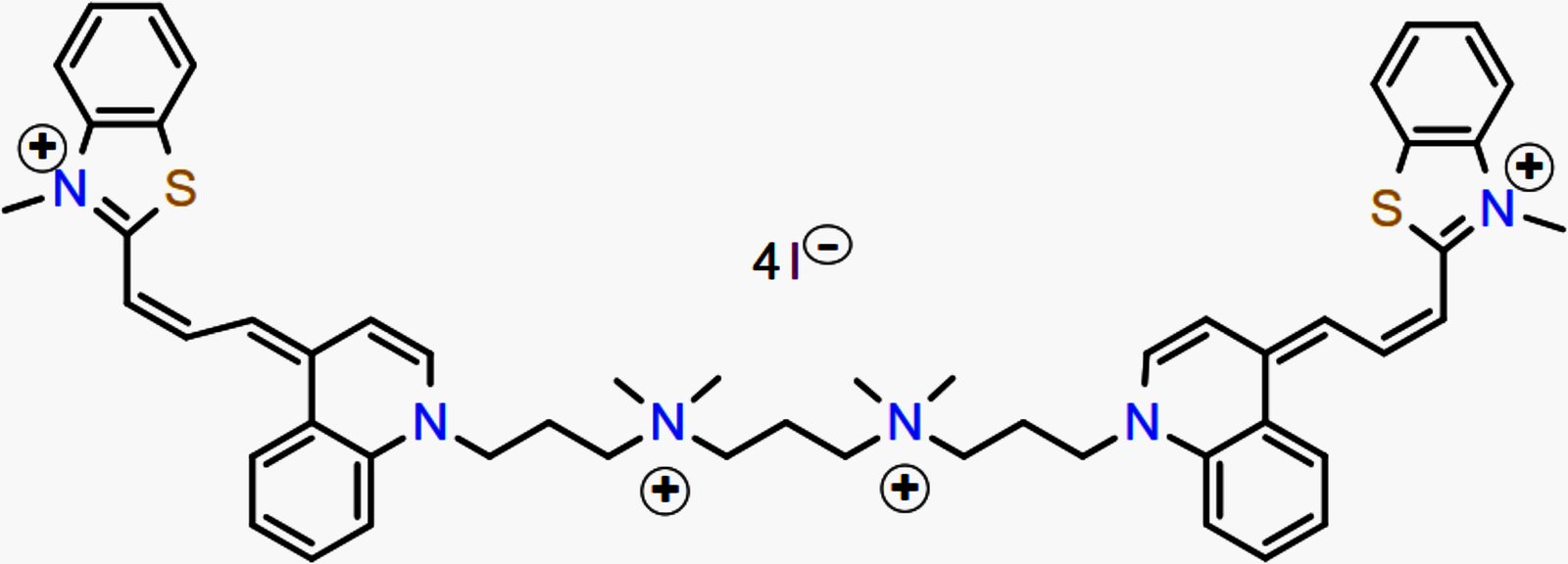	0.06	642/660	√	√	√	√	✘	√	Used to visualise eNA in biofilms [37]: detects DNase I -resistant eNA.
YOYO™-1	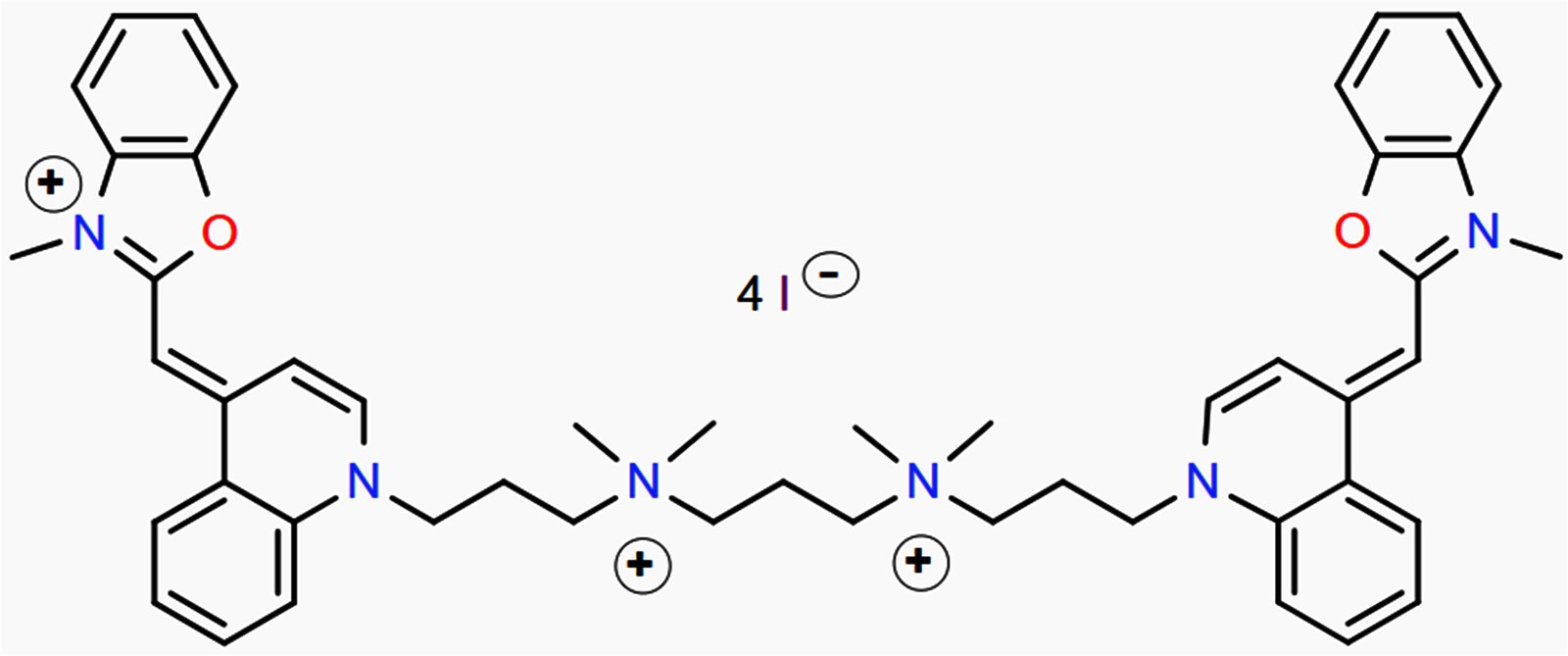	0.52	491/509	√	✘	√	√	✘	✘	Used for eNA visualisation and quantification in NETs [38,39].
YOYO™-3	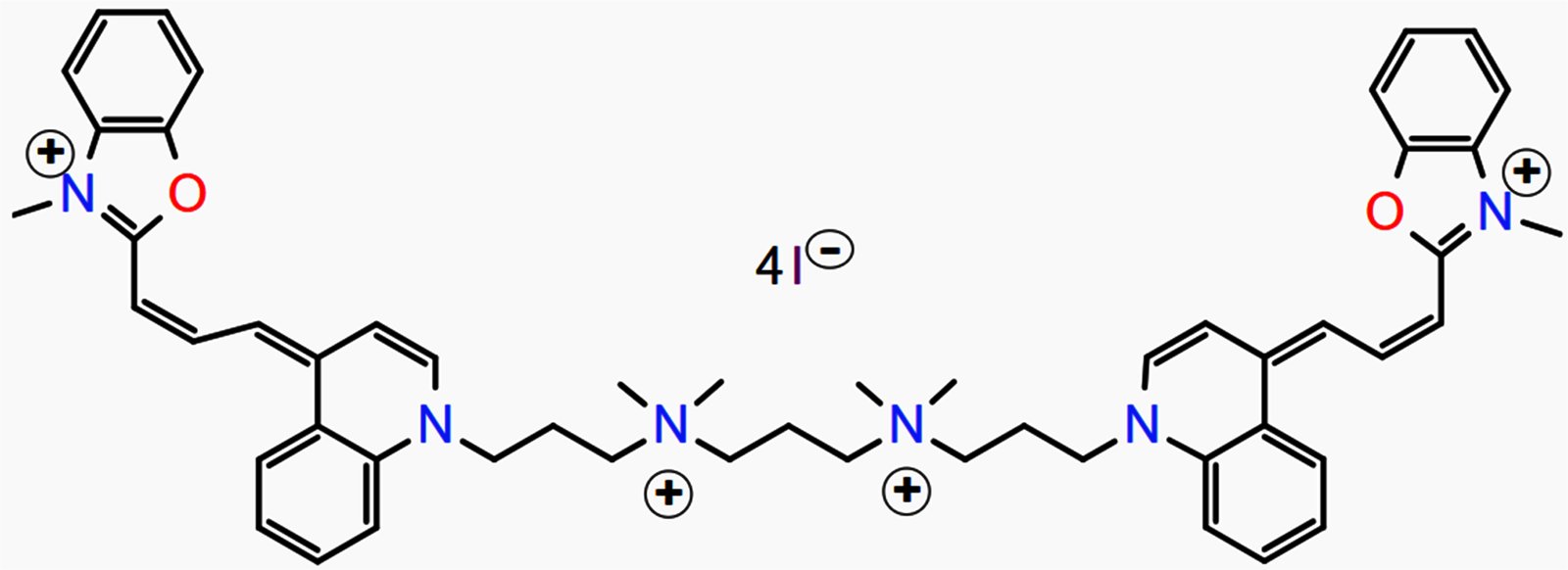	0.15	612/631	√	✘	√	✘	✘	√	Stains eNA around bacterial cells, similar to TOTO™-3 [30].
BOBO™-1	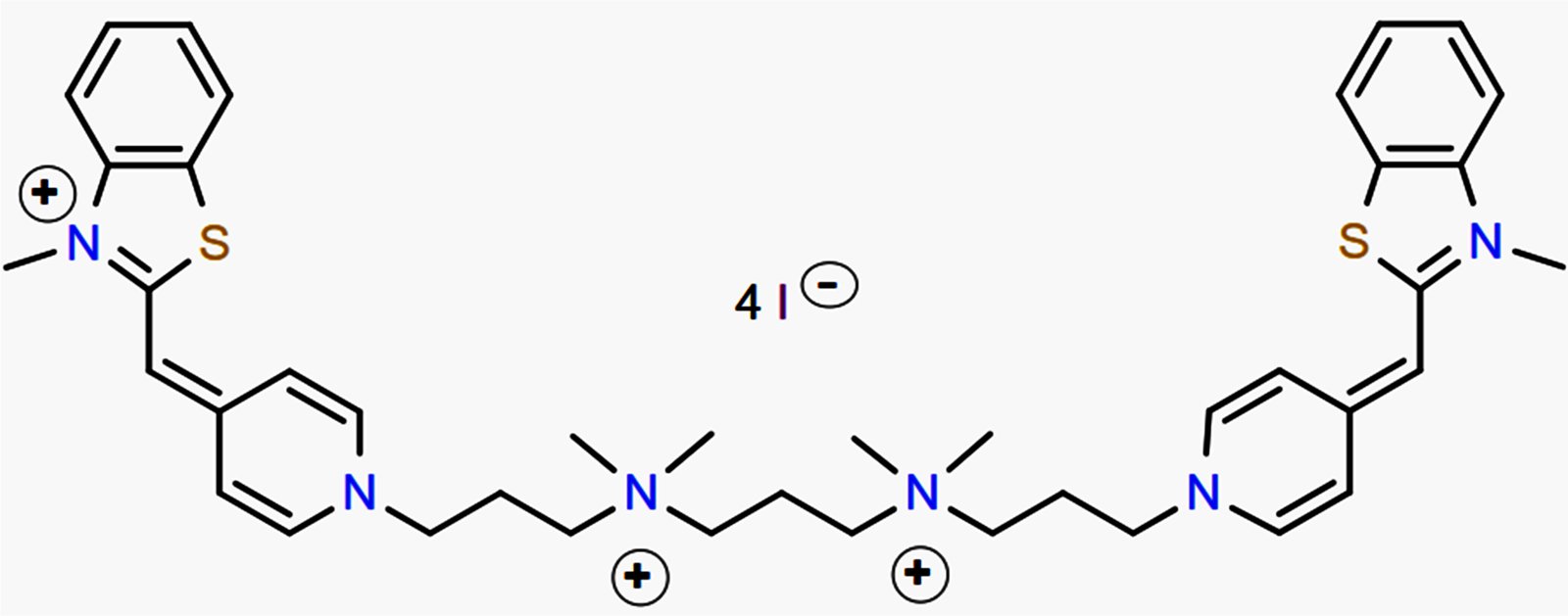	0.27	462/481	√	✘	✘	√	✘	✘	No data or research in biofilm available.
BOBO™-3	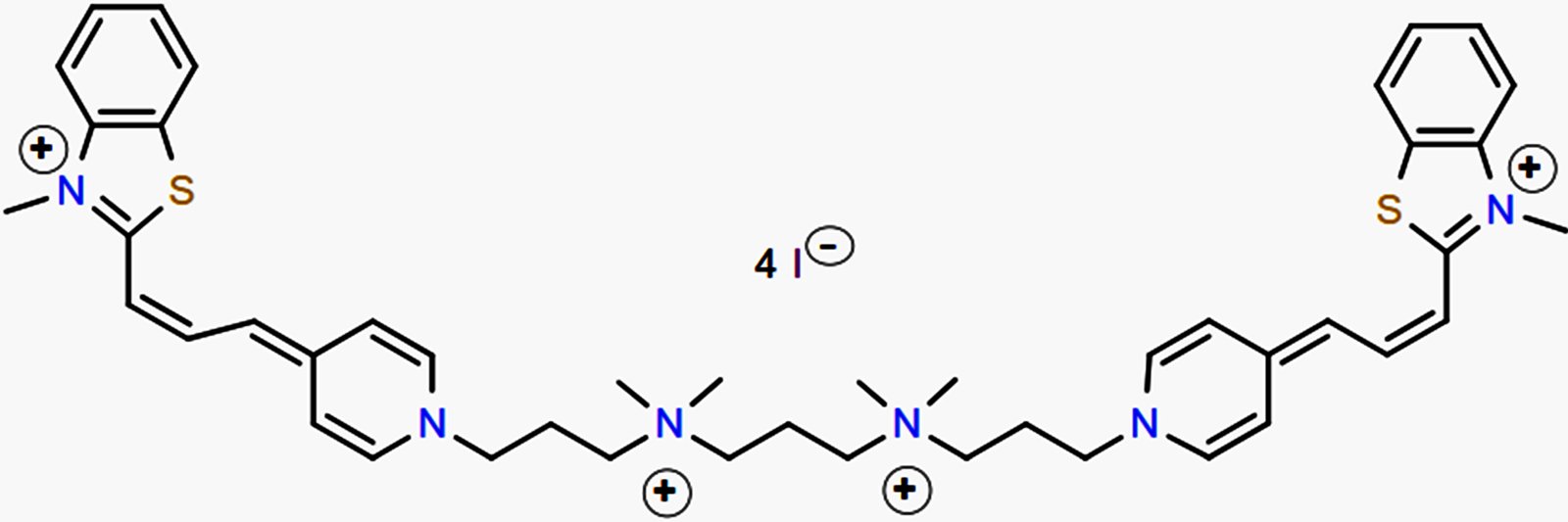	0.072-0.095	570/605	√	✘	√	√	✘	✘	Used to visualise eNA network in biofilm [40].
POPO™-1	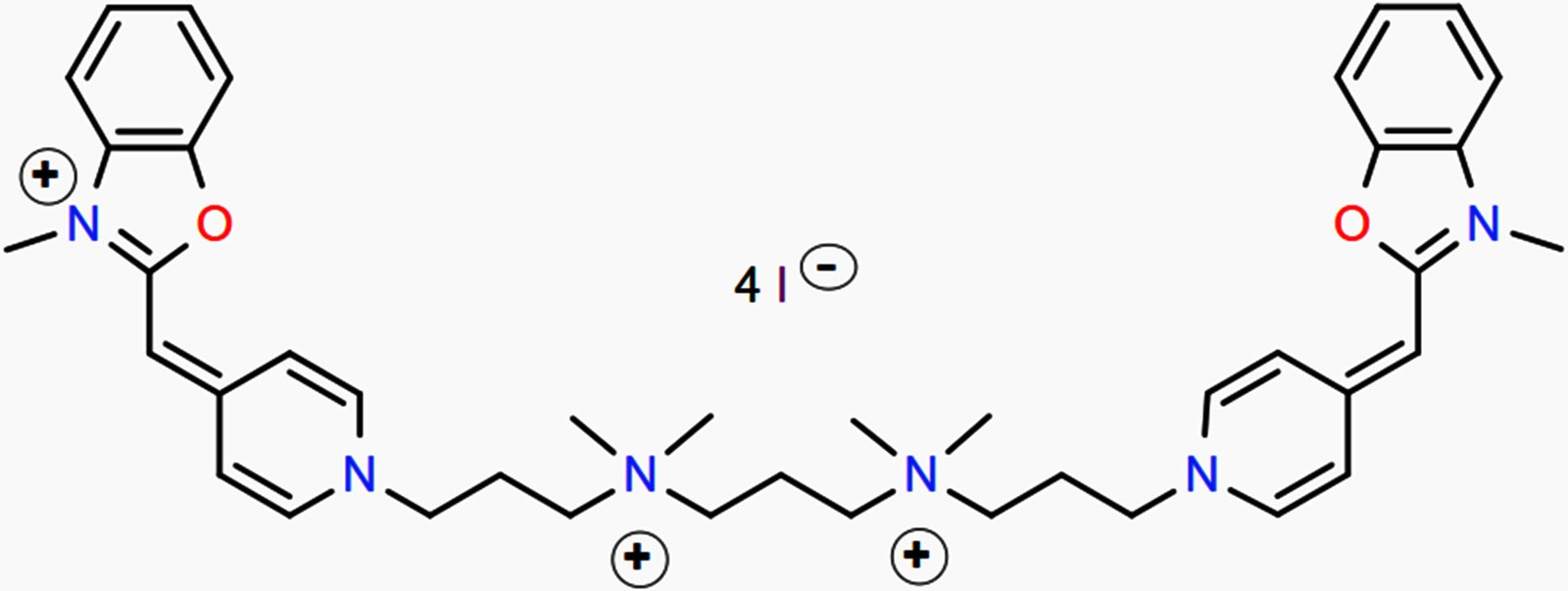	0.6	434/456	√	✘	√	√	✘	✘	Used to visualise eDNA in biofilm [41].
POPO™-3	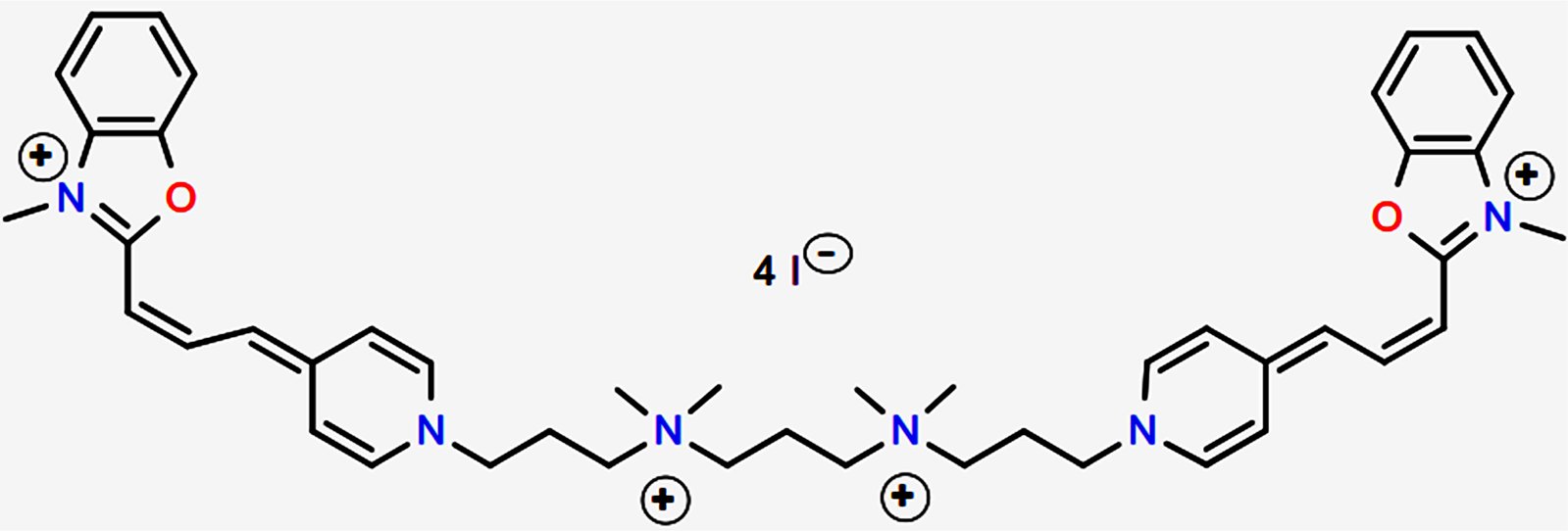	0.46	534/572	√	✘	√	√	✘	✘	Stains eNA around bacterial cells, similar to TOTO™-3 [30].
Cyanine monomers										
SYTOX™ series. Pro: High photostability. Con: Variable quantum yield										
SYTOX™ blue	Not available	-	440/480	√	-	-	√	-	-	No data or research in biofilm available.
SYTOX™ green	Not available	0.54	504/523	√	✘	✘	✘	✘	✘	Widely used in biofilm research, e.g. [42–44].
SYTOX™ orange	Not available	0.2	547/570	√	-	-	√	-	-	No data or research in biofilm available.
SYTOX™ red	Not available	0.2	640/658	√	√	√√	√	√	√√	The dye is provided as a 5 μM in DMSO and is unsuited for biofilm staining due to the toxicity of DMSO at the SYTOX™ Red working concentration.
SYTOX™ deep red	Not available	–	660/682	√	-	-	-	-	-	No data or research in biofilm available.
SYTOX™ AADvanced	Not available	–	546/647	√	-	-	√	-	-	No data or research in biofilm available.
Cyanine monomers (TOPRO series). Con: Can slowly enter cells.										
TOPRO-1	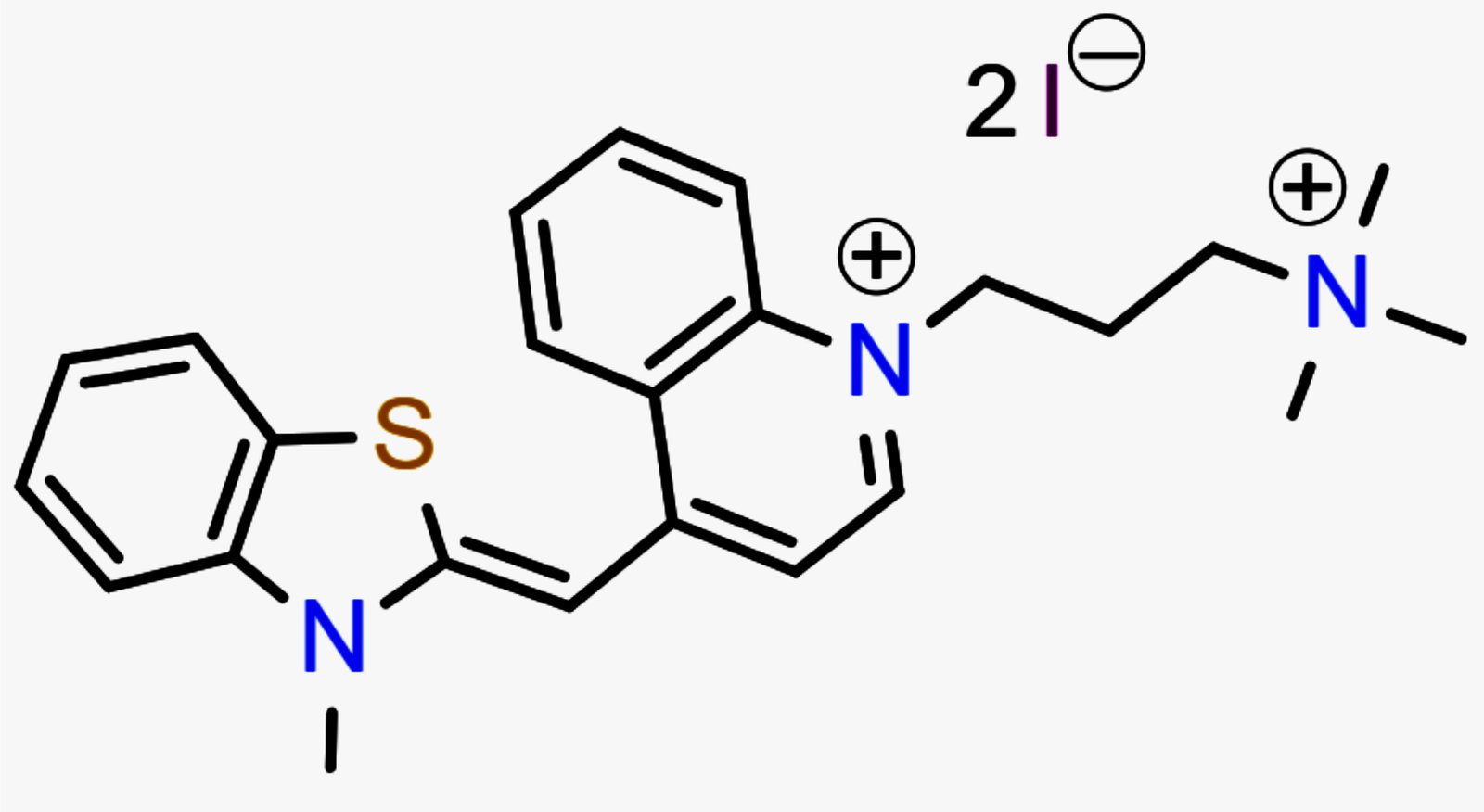	0.2	515/531	√	-	-	√	-	-	Claimed as membrane impermeable for assessment of viability in algae [45], but no data exist on biofilms.
TOPRO-3	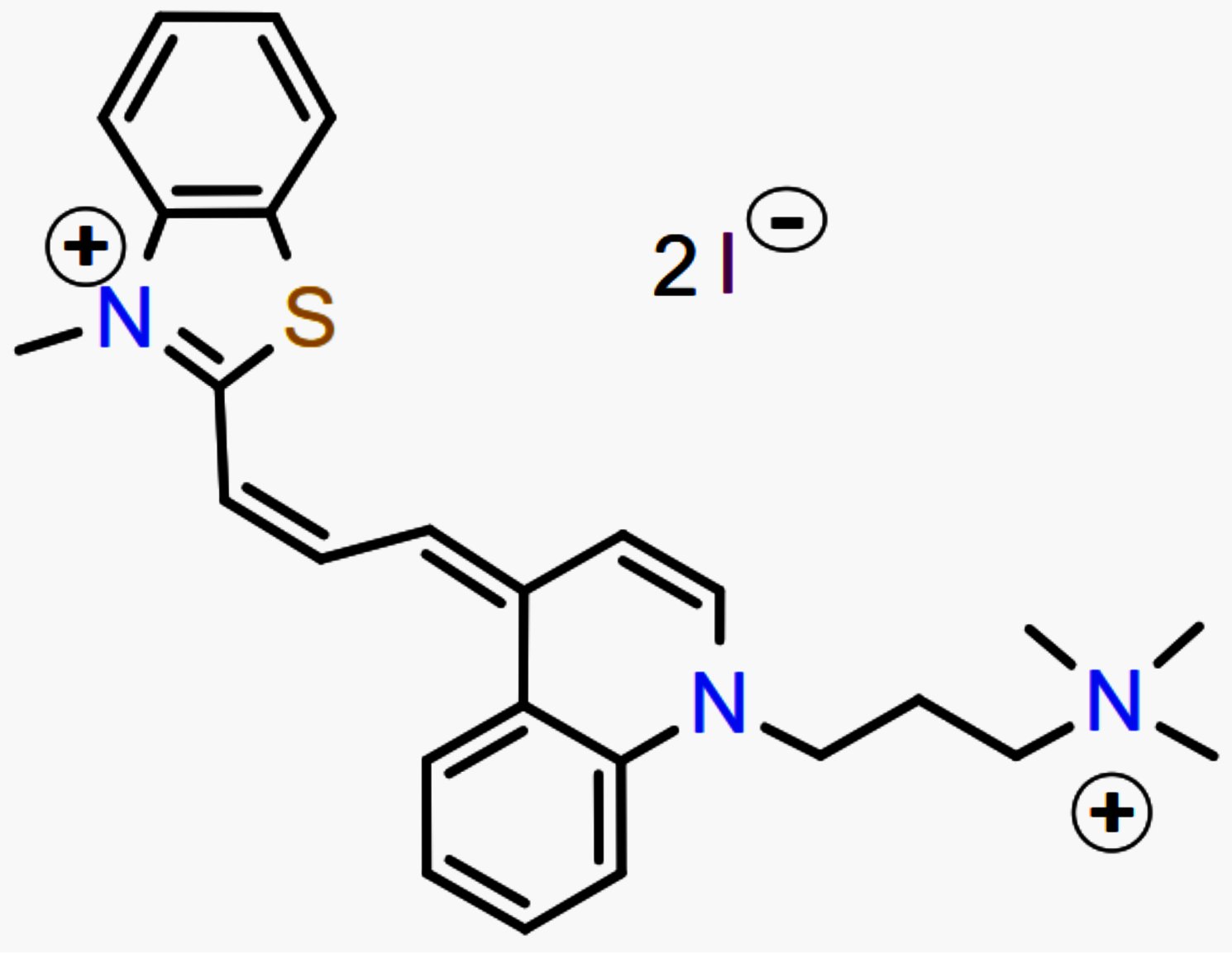	0.2	642/661	√	-	-	√	-	-	Several reports indicate that this dye is at least partially membrane-permeable [25,46,47].
Other dyes										
Propidium Iodide	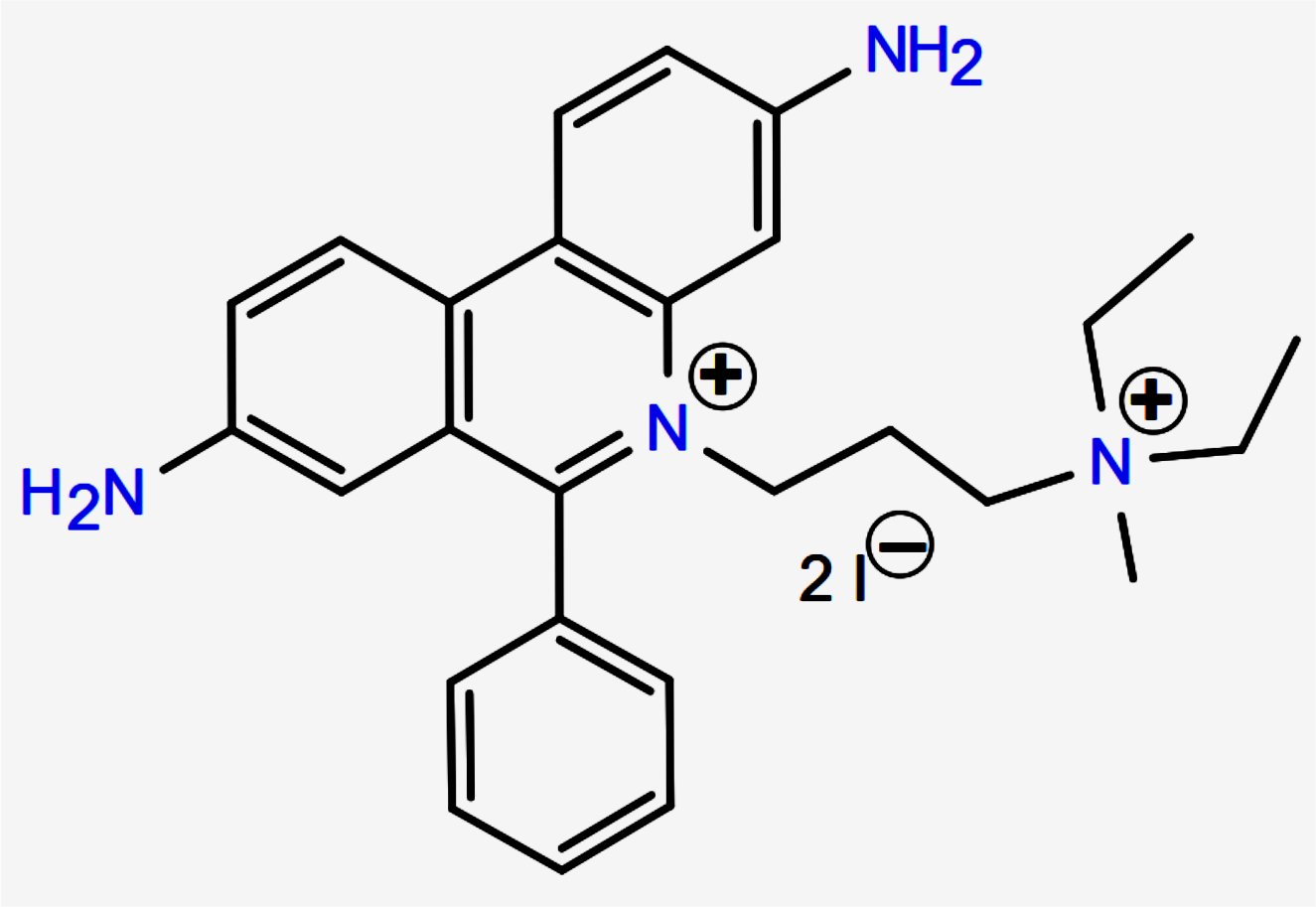	0.3–0.4	535/617	√	√	√	√√	√	√	Widely used in biofilm research to visualise eDNA [48,49,50].
DRAQ7	Not available	-	644/697	√	-	-	✘	-	-	Has high specificity for B-DNA and does not seem to bind to RNA according to manufacturer (Abcam). No data are available for biofilms.
DDAO	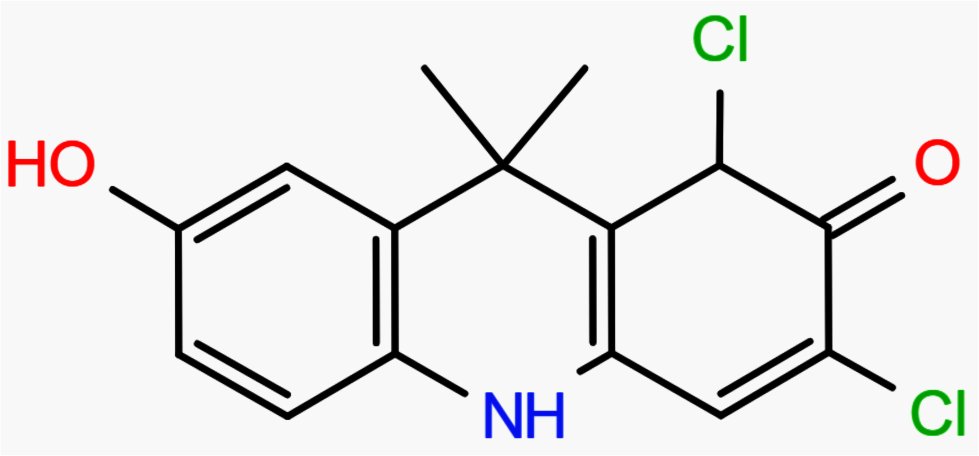	-	645/660	√	-	-	-	-	-	Previously used in biofilm research [51] but later studied showed that the dye slowly penetrates cells [25].
NMP	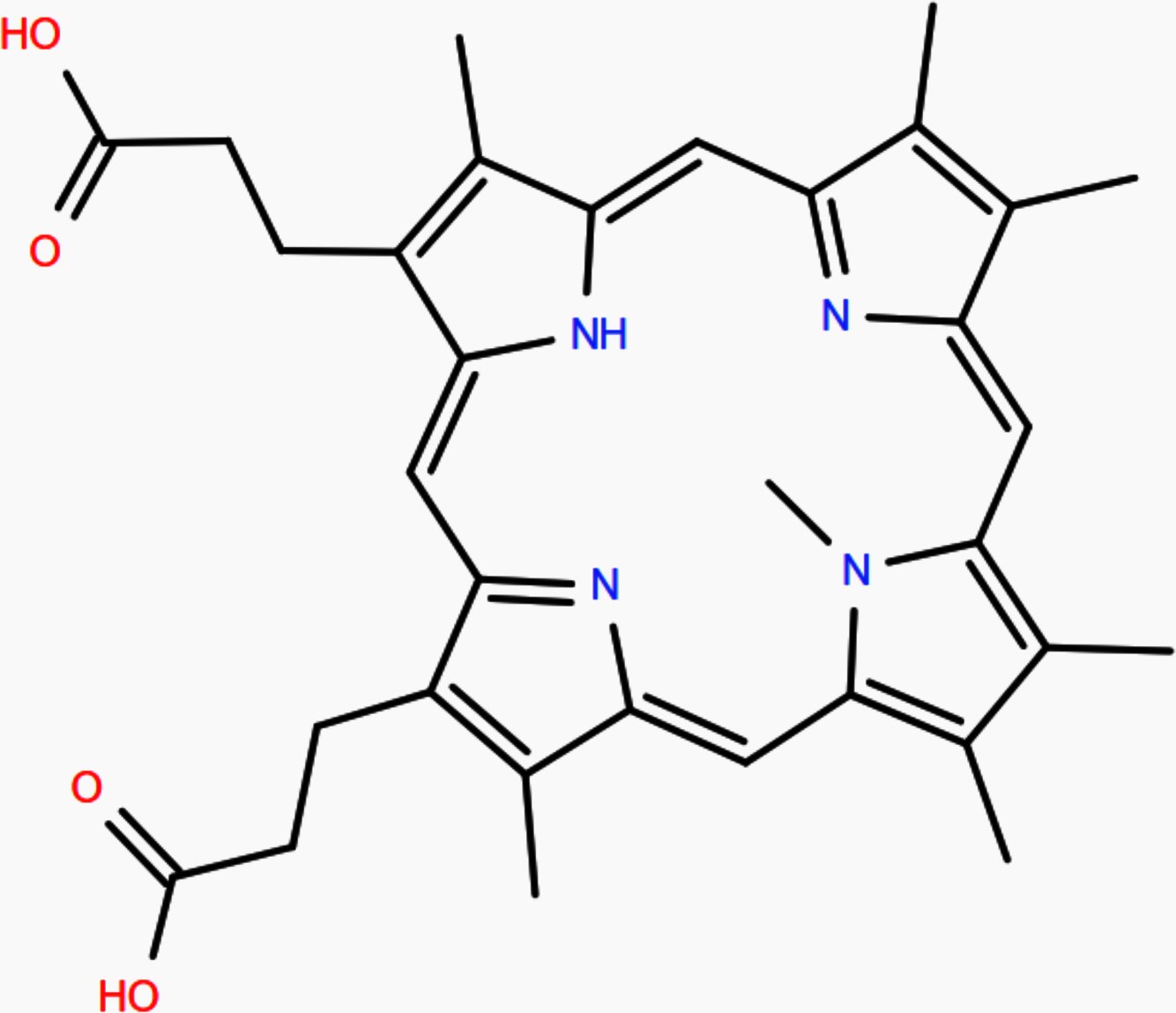	-	405/ 615	✘	✘	√	✘	✘	√	Specific for G4 but not very bright. It is therefore only suitable to visualise large amounts of G4 [13].

A prerequisite for using DNA-binding dyes to visualise eNA is that the dyes are membrane-impermeable. However, this aspect is not as binary as one might wish. Reports have, e.g., shown that high concentration and prolonged incubation with PI can lead to partial uptake [23]. Recent research suggests that PI-permeable membranes originate from certain stress responses but do not solely indicate dead cells [24]. DDAO and TOPRO dyes have also been reported as membrane-permeant [25].

If we consider brightness and photostability, the manufacturers provide information about the dyes’ quantum yield when complexed with B-DNA. Based on this information, the best dyes to stain B-DNA are the cyanine monomer SYTOX™ green, followed by cyanine dimers POPO™-1 > YOYO™-1 > POPO™-3 > TOTO™-1 and PI (Table 1). PI, which is frequently used in biofilm research with >10,000 total citations, stands out as being cheaper than the other dyes, which probably explains its popularity despite the lower quantum yield. However, it is highly carcinogenic, has low photostability, tends to precipitate [26], and can potentially interact with polysaccharides, as it has been reported to bind pectin in plant cell walls [27]. When experiments require high sensitivity and specificity, we therefore recommend selecting one of the brighter cyanine dyes.

DNA-binding dyes can differ in brightness when bound to AT versus GC-rich DNA sequences. In general, cyanine monomers do not display sequence specificity, while several cyanine dimers display preferential binding to AT-rich regions because the minor groove in these regions is wider and more accessible and favourable for electrostatic interactions with the cationic dimers. For example, BOBO™-3 displays higher fluorescence when binding to dAdT B-DNA compared with dGdC B-DNA, but the fluorescence lifetime was longer when bound to dGdC dsDNA [28]. TOTO™-1 has increased binding to dCdTdAdG-dCdTdAdG, which is explained by its ability to adapt to the base pair propeller twist of B-DNA to optimise stacking and hydrophobic interactions between the thymidine methyl group and the benzothiazole ring [29]. Thus, the GC content of NA and some sequence-specific variations in the fluorescence of the DNA-binding dyes must be considered when quantifying nucleic acids.

All the dyes listed in Table 1 were developed to bind B-DNA, and they are typically used for detection of purified DNA or for live/dead staining based on membrane permeability. The specificity for B-DNA versus other nucleic acid structures is irrelevant in that context. However, it is highly relevant to know the structure specificity when imaging eNA in complex biological samples like biofilms. With the recent discovery that non-canonical nucleic acid structures are abundant in biofilms, we must consider if these structures were previously overlooked for the simple reason that they are ‘invisible’ to standard DNA-binding dyes.

Sillesen et al. [30] examined a range of cyanine monomers and dimers by measuring fluorescence intensity from equimolar concentrations of oligos in different conformations. They observed that most dyes produced a strong fluorescence signal in B-DNA as well as A-RNA, but only a few detected G4-NA (BOBO™-3, YOYO™-3, TOTO™-3, and SYTOX™ Red) and Z-NA (TOTO™-3 and SYTOX™ Red). Hence, when TOTO™-1 is used in biofilm imaging, the absence of fluorescence only signifies the absence of B-DNA—not the other eNA structures. Interestingly, the commonly used PI displayed 4-fold higher intensity when bound to A-RNA compared with B-DNA, and it displayed some fluorescence in the non-canonical eNA, although not as bright.

We notice that these dyes fluoresce in the far-red spectrum. The cyanine dimers TOTO™-1/-3 differ dramatically in their specificity towards canonical vs. non-canonical NA [30]. This can be explained by the length of unsaturated chains, making the longer version of each dye (e.g., TOTO™-3) more hydrophobic than the shorter one (e.g., TOTO™-1). G4 binds a plethora of hydrophobic molecules [31], and the hydrophobic properties of the far-red dyes may contribute to increased G4 binding and fluorescence. The manufacturer does not disclose the molecular structure of SYTOX™ red, and it therefore remains an open question why this dye is so versatile in its interaction with nucleic acids due to its hydrophobicity.

**Figure 2:**
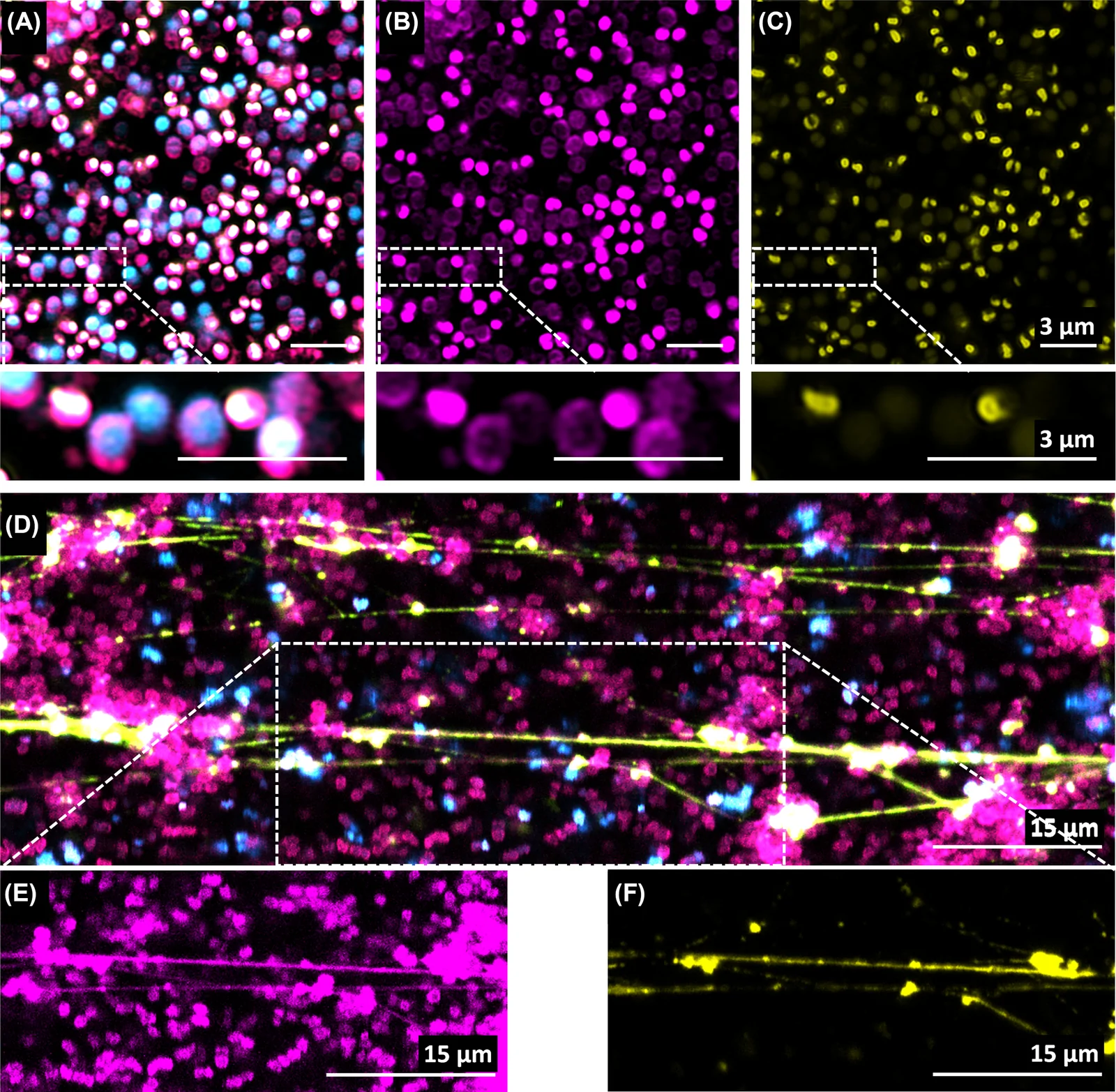
Visualisation of eNA in biofilms with DNA-binding dyes and antibodies (**A–C**) *Staphylococcus aureus* biofilms (prepared and labelled as described in [30]) stained with SYTO™41 (cyan), TOTO™-3 (magenta), and TOTO™-1 (yellow). The intracellular SYTO™41 dye visualises the location of viable bacteria, while TOTO™-1 and TOTO™-3 visualises eNA and dead cells. Notably, surface-associated eNA is only visible by TOTO™-3 staining and not by TOTO™-1, illustrating differences in their specificity. The insets show 3× magnified images. **(D–F)***Staphylococcus epidermidis* biofilm (prepared and labelled as described in [20]) stained with SYTO™41 (cyan) and TOTO™-3 (magenta) and immunolabelled with G4-specific BG4 antibody (yellow), visualising surface-associated eNA by TOTO™-3 and eNA strings labelled with both TOTO™-3 and BG4. The TOTO™-3 signal (**E**) and G4 immunolabeling (**F**) are shown individually to highlight that TOTO™-3 does bind to the eNA strings containing G4.

We encourage the use of multiple dyes in parallel when imaging eNA in biofilms to ensure detection of eNA beyond the canonical B-DNA form. The green-fluorescent dyes have higher quantum yield when binding to B-DNA (e.g., TOTO™-1, SYTOX™ Green), but the red and far-red dyes’ fluorescence also turns on in other eNA structures (TOTO™-3 and SYTOX™ Red). When used in combination, the different specificities can reveal the location of non-canonical eNA structures that preferentially bind e.g., TOTO™-3 over TOTO™-1, as seen in Figure 2A–C, where TOTO™-3 visualises surface-associated eNA while TOTO™-1 only visualises intracellular DNA in dead bacteria.

## Nucleic acid-binding antibodies

One of the challenges of using DNA-binding dyes is that they penetrate bacteria with damaged membranes, resulting in a very bright signal from dead bacteria in a specific field of view. Although a specific stain might be sensitive enough to detect small amounts of eNA in the extracellular matrix, the difference in fluorescence intensity for eDNA and dead cells is too large to capture both. Furthermore, as we discussed above, most DNA-binding dyes do not visualise non-canonical nucleic acid structures that play important roles in biofilms. Detection of eNA by fluorescence immunolabelling with structure-specific antibodies overcomes both of these challenges, as antibodies are structure-specific and do not penetrate bacteria with damaged membranes. Table 2 provides an overview of monoclonal antibodies (mAbs) available for this purpose.

**Table 2:** Antibodies used in the biofilm immunolabelling

Antibody	Epitope used for immunisation	eNA	Notes
27156 (clone 35I9)	Unknown	B-DNA	Widely applied to visualise eDNA in biofilms [18,58] and NETs [65].
Z22	Brominated DNA GCGCGC/CGCGCG	Z-NA (broad sequence)	Widely applied to visualise Z-DNA and Z-RNA in biofilms and in NETs [5,20,58,60,65]. It should be noted that it can induce Z-DNA formation [64].
Z-D11	GCGCGC/CGCGCG	Z-DNA (GC-rich)	High affinity and specificity for Z-DNA, showing cooperative binding, and does not induce Z-DNA formation [57]. No data are available for biofilms.
1H6	(TTTTGGGG)2	G4-DNA	Used to visualise G4-DNA in biofilm [19]. Specific for G4-DNA (not RNA) but also binds T-rich DNA [53].
BG4	A mix of G4 structures	G4-NA	Used to visualise G4-NA in biofilms [18,20]. Detects G4-DNA and -RNA and has a broader specificity to different G4 structures compared with 1H6 [53]. It has been reported to induce or stabilise G4-conformation [56].
iMab	CCC(TAACCC)3T	i-Motif	Detected in *S. epidermidis* biofilm [20].
Jel466	poly[d(Tm5C)]/poly[d(GA)].	Triplex	Detected in *S. epidermidis* biofilm [20].
J2	Reovirus RNA	A-RNA	Detected surface-associated A-RNA in *S. aureus* biofilm [30].
S9.6	Phage X174 replicative form	RNA/DNA hybrids	Detected RNA/DNA hybrids characteristic of R-loops in *Pseudomonas aeruginosa* biofilm [14].

Antibody mAb27156 (clone 35I9, Abcam) is the most widely used to selectively label double-stranded B-DNA (*K*_D_ 0.09 μM) and single-stranded DNA (*K*_D_ 0.71 μM) while having very weak reactivity with RNA, according to the manufacturer. Antibodies 1H6 and BG4 are used to selectively label G-quadruplex structures. 1H6 mostly labels parallel G4-DNA [52] although unspecific binding to non-G4 ssDNA is also possible [53]. The 1H6 mAb was first used by Seviour et al. for visualising G4-DNA in *P. aeruginosa* biofilms [19]. Furthermore, Seviour et al. used PI as a counterstain, demonstrating partial co-localisation of the 1H6 and PI signals. The 1H6 and BG4 mAbs were used by Minero *et al.* for visualising G4 in *S. epidermidis* biofilms [20]. In comparison with 1H6, BG4 has broader specificity, binding parallel, antiparallel, and hybrid G4-DNA, both intra- and intermolecular G4-DNA [54] as well as G4-RNA [55]. Importantly, studies suggest BG4 can recognise partially folded G4 structures and promote G4 stability as seen in, e.g., telomers [56]. Thus, BG4 immunolabelling generally detects more G4-NA, but it must be interpreted carefully, as the labelling procedure itself can introduce the structures we seek to detect. Minero et al. observed a more widespread distribution of BG4 compared with 1H6 in the eDNA of *S. epidermidis* biofilms [20], which either indicates that 1H6 does not visualise the most abundant G4 structures in the biofilm (e.g., G4-RNA) or that BG4 exacerbates the presence of G4 in the biofilm by stabilising partially folded G4 structures. These studies show that G4s are widespread in biofilms of bacteria with both high and low genomic GC content and may originate from both DNA and RNA. Based on the existing knowledge, we recommend using BG4 to identify the location of G4-DNA and G4-RNA structures and subsequently follow up with other methods for detecting G4 as discussed below.

Antibodies Z22 and Z-D11 selectively label Z-NA via sensing the alternating syn/anti backbone torsions and the phosphate zig-zag geometry [57]. Only Z22 is commercially available, and it has therefore been used to detect Z-DNA in a wide range of biofilms, including non-typable *Haemophilus influenzae, Escherichia coli, P. aeruginosa, Klebsiella pneumoniae* [58], *Salmonella enterica* [59], *S. epidermidis* [20], *S. aureus* [30], *Streptococcus mutans* [18], dental biofilms [18,60], clinical samples from airway infections [58], and even in biofilms in shower hoses [61]. Originally raised to detect polyGC epitope, Z22 mAb recognises the zig-zag conformation of Z-DNA [62] and not a particular epitope sequence [63]. Chin et al. [57] compared the high-resolution structure of Z22 and Z-D11 when bound to DNA, using cryo-EM. Both antibodies form filamentous trimers around DNA. Importantly, they showed that Z-D11 does not induce B- to Z-DNA transition under physiological conditions [57], while such a transition can be induced by Z22 [64]. Thus, Z22 immunolabelling results must be interpreted with caution as the labelling procedure itself can introduce the structures we seek to detect.

Antibodies Jel466 and iMab are used to selectively label triplex [66] and i-Motif NA [67], respectively. Together with other mAbs (1H6, BG4, and Z22), Jel466 and iMab labelled the web-like matrix in *S. epidermidis* biofilm, although their abundance was lower compared with mAbs staining G4 and Z-NA [20]. The presence of these eNA structures in the web-like eNA matrix suggests a common mechanism of their formation, although the ultimate role of triplexes and i-Motifs in biofilms is yet to be revealed.

Until recently, the presence of extracellular RNA (eRNA) in biofilms has been largely overlooked [30]. Application of RNA-specific antibodies may shed some light on the significance of eRNA in the future. The RNA-specific J2 antibody [68,69] was used to detect eRNA on the surface of *S. aureus* cells in biofilms [30], and the S9.6 antibody [70], specific for DNA/RNA hybrids in R-loops, also detected R-loops along eDNA strings in the wider matrix of *P. aeruginosa* biofilms [14]. Structural analysis of J2 and S9.6 shows, that they share a common recognition strategy [57], suggesting that they might be cross-reactive. Indeed, the R-loop-specific S9.6 antibody exhibits substantial cross-reactivity with A-RNA, but J2 does not recognise the DNA/RNA hybrids of R-loops [68].

In summary, immunolabelling can detect eNA with high sensitivity, and it can reveal non-canonical structures that are not stained by DNA-binding dyes. While this is a major advantage, some uncertainty about their specificity remains. Ultimately, it is important to perform thorough blocking and washing steps to avoid unspecific bindings of mAbs. Additionally, not much is known about the equilibrium between various eNA structures in biofilms and how these antibodies may affect such equilibrium. We recommend visualising eNA structures with multiple methods, e.g., by combining mAb with eNA-binding dyes as shown in Figure 2 D–F to assess if different methods corroborate the conclusions drawn from immunolabelling.

## Specific dyes and methods for detection of G4

Such corroboration of eNA structures can also be performed by including other methods for detecting non-canonical NA structures. For example, there are several alternative methods for visualising G4, such as *N*-methyl protoporphyrin (NMP), which was applied for real-time detection of extracellular G4-DNA synthesis in *S. oneidensis* planktonic cultures and biofilms, although the dye is not strictly extracellular [13]. Lund et al. have shown that a combination of DNA-binding dyes TOTO™-1 (green) and SYTO™60 (far-red) results in G4-specific fluorescence resonance energy transfer (FRET) and can be used for fast detection of extracellular G4-DNA in biofilms [36]. SYTO™60 binds to G4 with high affinity, and although SYTO™60 is a membrane-permeable dye, other studies have reported an extracellular signal from SYTO60 when used in combination with SYTOX™ green. Since SYTOX™ green is highly specific for B-DNA [30], selective staining by SYTO™60 might reflect the presence of extracellular G4 [43].

Another approach to detect G-quadruplexes is to exploit their peroxidase-like catalytic activity when complexing with hemin. This activity enables labelling by tyramide signal amplification, which is commonly used with horseradish peroxidase conjugated to, e.g., antibodies or DNA oligos for specific detection in biological samples. In this method, the peroxidase activity of G4/hemin is used to activate fluorescently labelled tyramide, which then becomes immobilised to tyrosine residues from nearby proteins, revealing the location of peroxidase activity in the biofilm when incubated with hemin and hydrogen peroxide. We note that an excess of hydrogen peroxide can lead to destruction of the hemin/G4 catalyst and therefore optimise reagent concentrations when using this method for the first time. We have shown that the tyramide signal amplification co-localises well with 1H6 labelling in biofilms [20]. The pros of this method are detection of specific catalytically active G4s in comparison with, e.g., unfolded G4s. The cons of this method are that not all G4s will have catalytic activity and can lead to false negatives.

## Telling the difference between DNA and RNA

With the recent discovery of eRNA in biofilms [14], we notice a significant methodological gap in the visualisation of eRNA in complex samples. While PI shows potential as an RNA screening tool, demonstrating a 4-fold increase in fluorescence when binding to A-RNA versus B-DNA [30], it still lacks absolute specificity. The A-RNA-specific antibody J2 may fill this gap. Furthermore, PolyA-tailing with labelled ATP has recently been introduced as a novel RNA visualisation tool [71], and the method has been successfully adapted for use in biofilms [30]. With the current need for an RNA visualisation technique, polyA-tailing is a promising tool as it allows for distinct RNA visualisation in complex samples.

Specific RNA sequences can also be detected in the extracellular matrix by single-molecule inexpensive fluorescent *in situ* hybridisation (smiFISH) [72], which was used recently to show eRNA contains a high abundance of a specific mRNA (*lasB*) in *P. aeruginosa* biofilm [73]. With the advent of CLASI-FISH [74], a broad spectrum of RNA sequences could be detected simultaneously by smiFISH.

## Conclusion

The discovery of non-canonical extracellular nucleic acid structures in biofilms represents a paradigm shift in our understanding of the biofilm matrix. Until recently, eNA were primarily defined as extracellular DNA in the canonical B-form, inadvertently overlooking both eRNA and non-canonical structures such as Z-DNA, G-quadruplexes, i-motifs, and triplexes. This oversight stemmed partly from methodological limitations—propidium iodide and TOTO™-1, the most widely used dyes, exhibit strong structural preferences for A-RNA and B-DNA, respectively, rendering other eNA structures effectively invisible.

These detection biases have important implications. The persistence of propidium iodide fluorescence in DNase I-treated biofilms may reflect selective staining of eRNA rather than incomplete DNA degradation. Similarly, absence of TOTO™-1 signal indicates absence of B-DNA specifically, not absence of eNA. To capture the full structural diversity, we recommend combining far-red fluorescent dyes with TOTO™-1, adjusting CLSM histogram settings to detect dim structures, and validating findings with structure-specific immunolabelling using commercial antibodies (Z22, BG4, 1H6, iMab, Jel466, and J2). Alternative methods including G4/hemin-catalysed tyramide signal amplification and polyA-tailing offer complementary approaches that reveal functional states.

Beyond structural diversity, non-canonical eNA possess emergent functional properties. G-quadruplexes complexed with hemin form catalytically active DNAzymes and serve as conduits for extracellular electron transfer, enabling metabolic activity in anoxic biofilm regions. Z-DNA and G-quadruplexes resist mammalian DNase I degradation, explaining why this enzyme fails to disperse mature biofilms. These properties have significant therapeutic implications: enzymatic targeting with micrococcal nuclease (for G-quadruplexes) or S1 nuclease (for Z-DNA) represents a promising avenue for biofilm control, requiring structure-specific detection tools for rational therapeutic design.

## Summary

- eNA in biofilms comprise diverse structural forms beyond canonical B-DNA.
- Widely used DNA-binding dyes exhibit strong structural biases that have obscured the true diversity of eNA.
- Structure-specific detection requires complementary methods combining fluorescent dyes with immunolabelling.
- Immunolabelling results must be interpreted with caution, as some antibodies can induce and stabilise the very structures they detect.
- Non-canonical eNA structures represent promising therapeutic targets for biofilm infections, and visualising these structures in infections can provide a foundation for developing therapeutics that target the biofilm matrix via these robust structures.

## Open Access

Open access for this article was enabled by the participation of Aarhus Universitet in an all-inclusive Read & Publish agreement with Portland Press and the Biochemical Society under an agreement with Royal Danish Library.

## Abbreviations

eDNA: extracellular DNA
eNA: extracellular nucleic acids
eRNA: extracellular RNA
FRET: fluorescence resonance energy transfer
mAbs: monoclonal antibodies
NMP: *N*-methyl protoporphyrin
PI: propidium iodide
smiFISH: single-molecule inexpensive fluorescent in situ hybridisation
